# Movement behavior patterns composition remains stable, but individuals change their movement behavior pattern over time in people with a first-ever stroke

**DOI:** 10.1186/s11556-022-00290-4

**Published:** 2022-04-22

**Authors:** Patricia J. van der Laag, Roderick Wondergem, Martijn F. Pisters

**Affiliations:** 1 Physical Therapy Sciences, Program in Clinical Health Sciences, Utrecht University, Utrecht, the Netherlands; 2 Center for Physical Therapy Research and Innovation in Primary Care, Julius Health Care Centers, Utrecht, the Netherlands; 3 Department of Rehabilitation, Physical Therapy Science and Sport, Brain Center, University Medical Center, Utrecht, the Netherlands; 4 Research Group Empowering Healthy Behaviour, Department of Health Innovations and Technology, Fontys University of Applied Sciences, Eindhoven, the Netherlands

**Keywords:** Movement behavior, Physical activity, Secondary prevention, Sedentary behavior, Stroke

## Abstract

**Background:**

Movement behaviors (i.e., physical activity levels, sedentary behavior) in people with stroke are not self-contained but cluster in patterns. Recent research identified three commonly distinct movement behavior patterns in people with stroke. However, it remains unknown if movement behavior patterns remain stable and if individuals change in movement behavior pattern over time.

**Objectives:**

1) To investigate the stability of the composition of movement behavior patterns over time, and 2) determine if individuals change their movement behavior resulting in allocation to another movement behavior pattern within the first two years after discharge to home in people with a first-ever stroke.

**Methods:**

Accelerometer data of 200 people with stroke of the RISE-cohort study were analyzed. Ten movement behavior variables were compressed using Principal Componence Analysis and K-means clustering was used to identify movement behavior patterns at three weeks, six months, one year, and two years after home discharge. The stability of the components within movement behavior patterns was investigated. Frequencies of individuals’ movement behavior pattern and changes in movement behavior pattern allocation were objectified.

**Results:**

The composition of the movement behavior patterns at discharge did not change over time. At baseline, there were 22% *sedentary exercisers* (active/sedentary), 45% *sedentary movers* (inactive/sedentary) and 33% *sedentary prolongers* (inactive/highly sedentary). Thirty-five percent of the stroke survivors allocated to another movement behavior pattern within the first two years, of whom 63% deteriorated to a movement behavior pattern with higher health risks. After two years there were, 19% *sedentary exercisers*, 42% *sedentary movers,* and 39% *sedentary prolongers.*

**Conclusions:**

The composition of movement behavior patterns remains stable over time. However, individuals change their movement behavior. Significantly more people allocated to a movement behavior pattern with higher health risks. The increase of people allocated to sedentary movers and sedentary prolongers is of great concern. It underlines the importance of improving or maintaining healthy movement behavior to prevent future health risks after stroke.

**Supplementary Information:**

The online version contains supplementary material available at 10.1186/s11556-022-00290-4.

## Background

Stroke is one of the largest causes of mortality and long-term disability worldwide [1]. Worldwide, about 80 million [2] people live with the consequences of a stroke [3]. People with stroke are at high risk for recurrent stroke, cardiovascular diseases, and premature mortality [1, 3–6]. One of the key modifiable risk factors to prevent secondary health risks after stroke is decreasing sedentary time and improving the time spend in physical activity (PA) [6–8]. Although PA benefits are well recognized, the levels of PA in people with stroke are still half of those of healthy older adults [8, 9]. Moreover, people with stroke are highly sedentary [6, 7, 10–12].

To prevent recurrent stroke(s) or other cardiovascular events, research suggests targeting all components of movement behavior [13]. Movement behavior consists of sedentary behavior (SB) and all PA levels (i.e., light, moderate, and vigorous). Although the single aspects of movement behavior are independently associated with health risks, they are not self-contained and cluster in patterns [14, 15]. Moreover, one behavior’s health benefits could be inadequate to compensate for the health risks of one other aspect [11, 16]. Therefore, there is growing interest in an optimal distribution of SB and PA levels and health outcomes in people with stroke during waking hours.

In a cross-sectional study, we recently identified three distinct movement behavior patterns in people with a first-ever stroke [6]. The most active group, “*sedentary exercisers”,* showed sufficient amounts of moderate to vigorous physical activity (MVPA) (1.4 h/day). Nevertheless, they were still sedentary for 63% of their waking hours in relatively short bouts. “*Sedentary movers*” showed similar amounts and interruption of SB as the “*sedentary exercisers*”. However, they showed low amounts of MVPA (0.4 h/day). “*Sedentary prolongers*” showed considerable amounts of SB (78%) in long prolonged bouts and low amounts of MVPA (0.4 h/day) [6].

Studies investigating the course of movement behavior aspects up to the first year after stroke have used average group data and found no changes over time [4, 11, 17, 18]. However, recovery after stroke is not a one-size-fits-all principle; it is characterized by individual patterns [19]. Therefore, it is hypothesized that the composition of movement behavior patterns remains stable over time. Nevertheless, individuals probably might change their movement behavior pattern.

Insight into movement behavior patterns in people with stroke ultimately enables more targeted and personalized secondary prevention in people with unhealthy movement behavior. Before developing interventions, we need to know if movement behavior patterns’ composition remains stable over time and if individuals change their movement behavior pattern. This will enable health care professionals to identify the right people with an unhealthy movement behavior pattern and offer more personalized trajectories. Therefore, the aims of this study were; 1) to investigate the stability of the composition of movement behavior patterns over time, and 2) determine if individuals change their movement behavior resulting in allocation to another movement behavior pattern within the first two years after discharge to home in people with a first-ever stroke.

## Methods

### Design and setting

This study is part of a multi-center longitudinal prospective cohort study (RISE-cohort study) [6], which followed people with a first-ever stroke for two years. Between February 2015 and April 2017, participants with a first-ever stroke were recruited from four stroke-units of hospitals in the Netherlands.

Participants were deemed eligible if they were: 1) clinically confirmed with a first-ever stoke, 2) expected to be discharged to a home setting, 3) independent in activities of daily living before onset (Barthel Index > 18), 4) aged 18 years and older at time of stroke, 5) able to maintain a conversation (> 4 Utrecht Communication Assessment) and 6) able to walk at least with supervision at the time of discharge to home (Functional Ambulation Categories ≥3). Participants were excluded if they had a life expectancy of less than two years and insufficient Dutch-speaking and reading skills to understand and follow instructions. The medical ethical committee of the University of Utrecht approved the RISE study (study number 14/76). All participants gave written informed consent.

Participants were visited at home three weeks, six months, one year, and two years after discharge to home from a hospital or inpatient (geriatric) rehabilitation. Participants wore an accelerometer for two consecutive weeks on all four time points during waking hours and land-based activities. Three weeks after discharge, questionnaires and physical tests were conducted. Demographic data and stroke characteristics were retrieved from the participants’ patient files (additional file 1).

### Variables

Movement behavior was objectified by an accelerometer (Activ8) [20]. The Activ8 is a valid tool [21, 22] for measuring the energy expenditure and time spent in lying/sitting, standing, walking, running, cycling, and non-wear [20]. For an optimal recording of SB and PA levels, data were considered valid if at least seven days, ten hours were recorded [6, 23, 24]. As in the research of Wondergem et al. [6] ten movement behavior variables were retrieved from the data; mean time spent in sedentary behavior, light physical activity (LPA) and MVPA, mean time spent in sedentary bouts ≥5 min, ≥30 min, and ≥ 60 min, mean time MVPA in bouts ≥10 min, weighted median sedentary bout length, maximum sedentary bout length, and fragmentation index [6]. The weighted median sedentary bout is the bout length corresponding to 50% of the total sedentary time, ordered from shortest to longest. The lower the weighted median bout length, the more interruption in SB. Fragmentation index is the ratio of the number of sedentary bouts ≥5 min divided by total sedentary time. The higher the fragmentation index, the higher amount of interrupted SB.

Demographic characteristics included age at the time of stroke, sex, discharge destination (hospital, inpatient (geriatric) rehabilitation), and living status (alone or not alone). Physical Activity Assessment scale assessed pre-stroke MVPA. A score ≥ 4 indicates a sufficient amount of MVPA [25]. Social Support List measured the patients’ experience of social support [26, 27]. Twelve items are scored on a four-point Likert scale. A higher score (range 12–48) indicates more social support [26, 27]. National Institutes of Health Stroke Scale measured the severity of stroke [28, 29]. Eleven items score the stroke severity. A higher score (range 0–42) indicates more severe stroke symptoms [29]. The Cumulative Illness Rating Scale assessed comorbidity. Thirteen categories with disorders are scored. A higher score (range 0–52) indicates more serious comorbidity [30].

Motor function was assessed with the Motricity Index [31], which tests random movement activity and maximal isometric strength [31]. Five Meters Walk Test measured walking performance. The calculated walking speed (m/s) indicates ambulation type [32]. Berg Balance Test tested balance. A higher score (range 0–56), indicates a better balance [33–35]. Functional status was assessed with the subdomain physical functioning of the Stroke Impact Scale 3.0 [36–38]. The items assess strength, activities in daily living, mobility, and hand function. A standardized score is calculated for all domains. A higher score (range 0–100) indicates better self-perceived physical functioning [36].

Cognitive functioning was assessed with the Montreal Cognitive Assessment. A total score < 26 (range 0–30) indicates cognitive impairment [39, 40]. Hospital Anxiety and Depression Scale measured anxiety and depression [32, 41]. Fourteen items, seven about anxiety and seven about depression, are scored on a four-point Likert scale. A higher score on each subscale (range 0–21) indicates more complaints [42]. Checklist Individual Strength-Fatigue subscale evaluated fatigue. The total score was dichotomized, > 40 (range 8–56) indicates severe fatigue [32, 43]. Self-Efficacy for Symptom Management Scale assessed self-efficacy. The total score was dichotomized, < 115 (range 13–130) indicates low to moderate self-efficacy [32, 44].

### Data analysis

Data were analyzed using SPSS software [45]. Missing data were found missing at random. Therefore, a multivariate imputation by chained equations (MICE) procedure with 50 iterations was used to create a complete dataset [46]. Based on a correlation matrix, variables were entered into the model. A total of five imputed datasets were created and pooled into one set according to Rubin’s rule [47]. Hereafter, the normality of the data was checked by QQ-plots and histograms.

Principal Componence Analysis (PCA) compressed ten movement behavior variables into three components. The explained variance of three components and, z-scores were calculated for the compressed components on all four time points. Hereafter, three components were included for K-means clustering to identify three movement behavior patterns on all four time points. K-means clustering assumes each participant belongs to one group [48]. First, data points are randomly assigned to a cluster [48]. Hereafter, centers of the groups will be calculated, and individuals will be reassigned to a movement behavior pattern based on the center of the group and data point [48, 49].

Normally distributed movement behavior variables were presented as mean ± standard deviation or mean [95% confidence interval]. Non-normally distributed movement behavior variables were presented as median and Interquartile Range (IQR). Descriptive variables were presented as median (IQR) or absolute number (N) and percentage (%).

The stability of the composition of movement behavior patterns was checked by comparing scatterplots of the components’ distribution per pattern at baseline with the patterns at follow-up assessments. Hereafter, repeated measurement Anova was used to test the null hypothesis of equal means of the distance between cluster centers on all four time points. The composition of movement behavior patterns was considered stable when the distribution of components was similar, and the mean difference from cluster center was equal on all four time points.

Variables between patterns were analyzed with One way Anova or with Kruskal Wallis test if variables were not normally distributed. Chi-square test was used for categorical variables. Post hoc analysis were performed with Bonferroni correction for multiple comparisons.

Because of the partially paired and unpaired groups over time, differences within movement behavior variables over time were compared per pattern with Linear mixed model. Non-normally distributed movement behavior variables were transformed by square root to enter the model. Hereafter, variables were converted back to present median (IQR).

Finally, the proportion of individual’s movement behavior pattern and the frequency of individuals’ changes their movement behavior patterns were calculated on all four time points.

## Results

**Fig. 1:**
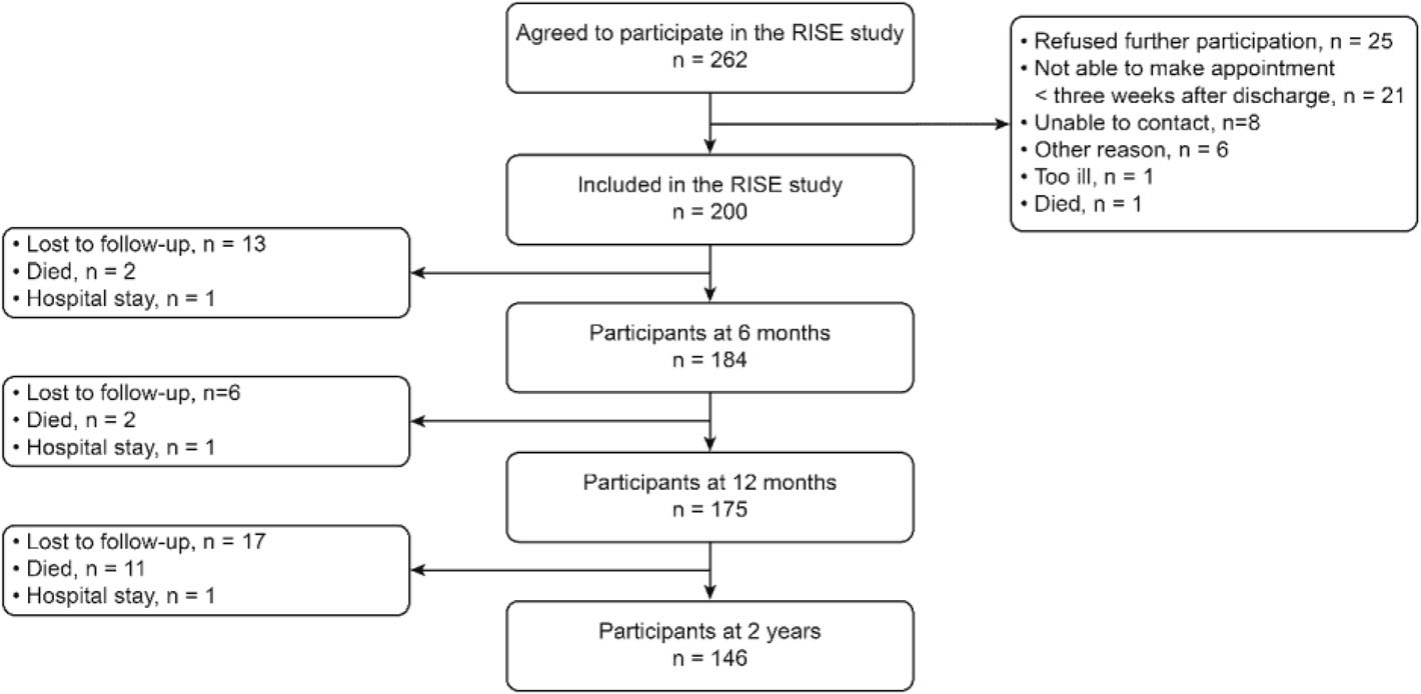
Flow diagram of sample generation

In total, 200 participants were included for analysis (Fig. 1). Missing data of 54 participants were imputed.

Participants’ characteristics are presented in Table 1. The mean age at onset of stroke was 67.8 years, 64% were male, and 73.5% of the participants were discharged directly to the home setting. At baseline, the mean wear-time was 13.7 h per day. Mean SB, LPA, and MVPA per day were respectively 9.3 h (67.8%), 3.8 h (27.6%), and 0.6 h (4.7%). Weighted median sedentary bout length was 22.5 min per day, and MVPA accumulated in bouts of ≥10 min for 0.13 h per day.

**Table 1:** Participants’ baseline characteristics and characteristics presented for the overall group and per movement behavior pattern

	Total group *N* = 200	Sedentary exercisers *N* = 44	Sedentary movers *N* = 90	Sedentary prolongers *N* = 66
Age	67.8 ± 11.5	62.6 ± 11.2†‡	69.2 ± 11.6*	69.3 ± 10.8*
Sex (male)	128 (64%)	35 (80%)†	51 (57%)*	42 (63%)
Discharge destination				
Hospital	147 (74%)	35 (80%)	68 (76%)	44 (67%)
IPR	24 (12%)	4 (9%)	10 (11%)	10 (15%)
IPGR	29 (15%)	5 (11%)	12 (13%)	12 (18%)
Living status (alone)	48 (24%)	12 (27%)	25 (28%)	11 (17%)
Pre stroke MVPA (PAA)	4 (2.0)	5 (2.0) ‡	4 (2.0)	4 (2.0)*
Social support (SSL)	31 (8.0)	32 (7.7)	31.7 (9.3)	30 (8.0)
Stroke severity (NIHSS)	3 (4.0)	2 (5.0)	2 (4.0)	3 (4.3)
Comorbidity (CIRS)	3 (4.0)	1.5 (4.0) †‡	3 (3.0)*	3 (5.0)*
Motor function (MI)				
Upper extremity	100 (0)	100 (0)	100 (8.0)	100.0 (8.0)
Lower extremity	100 (0)	100 (0.0) ‡	100.0 (0.0)	100.0 (2.25)*
Walk performance (5MWT)	5.0 (1.8)	4.7 (0.8) †‡	5.3 (2.1)*	5.4 (3.2)*
Balance (BBS)	55.0 (6.0)	56.0 (1.0) †‡	54.0 (7.0)*	53.0 (8.0)*
Functional status (SIS)	90.6 (21.9)	96.4 (9.1) †‡	88.0 (25.3)*	85.9 (25.3)*
Cognitive functioning (MOCA)	25 (5.0)	25 (4.0)	25 (5.0)	24.5 (5.3)
Anxiety	5 (5.8)	5.0 (6.8)	6.0 (6.0)	5.0 (4.0)
Depression (HADS)	5.0 (6.0)	3.5 (6.0)	5.0 (6.0)	5.0 (4.3)
Fatique (CIS-F) (severe)	40 (20%)	5 (11%)	18 (20%)	17 (26%)
Self-efficacy (SESx) (low-moderate)	170 (85%)	37 (84%)	73 (81%)	60 (91%)
Sedentary time (hours/day)	9.3 ± 1.8	9.0 ± 1.6‡	8.4 ± 1.5‡	10.6 ± 1.40*†
SB percentage	67.8	63.6‡	63.1‡	77.1*†
LPA time (hours/day)	3.8 ± 1.5	3.8 ± 1.2†‡	4.6 ± 1.5*‡	2.8 ± 0.8*†
LPA percentage	27.6	26.9†‡	33.6*‡	20.0*†
MVPA time (hours/day)	0.6 (0.7)	1.3 (0.3) †‡	0.4 (0.5)*	0.4 (0.5)*
MVPA percentage	3.9	9.2†‡	2.9*	2.6*
MVPA bouts ≥10 min. (hours/day)	0.1 (0.3)	0.6 (0.4) †‡	0.1 (0.2)*	0.1 (0.2)*
Weartime	13.7 ± 1.4	14.1 ± 1.5^†^	13.4 ± 1.3^*^	13.8 ± 1.5

Data is presented as mean ± SD, median (IQR) or n (%).

*IPR* inpatient rehabilitation, *IPGR* inpatient geriatric rehabilitation, *PAA* Physical Activity Assessment scale, *SSL* Social Support List, *NIHSS* National Institutes of Health Stroke Scale, *CIRS* Cumulative Illness Rating Scale, *MI* Motricity Index, *5MWT* Five Meter Walk Test, *BBS* Berg Balance Scale, *SIS* Stroke Impact Scale 3.0, *MOCA* Montreal Cognitive Assessment, *HADS* Hospital Anxiety and Depression Scale, *CIS-F* Checklist Individual Strength-Fatigue subscale, *SESx* Self-Efficacy for Symptom management scale, *LPA* light physical activity, *MVPA* moderate-vigorous physical activity.

* = significant differences with sedentary exercisers

† = significant differences with sedentary movers.

‡ = significant. Differences with sedentary prolongers.

### Stability of movement behavior patterns composition

Through PCA, three components were compressed for all four time points. Movement behavior variables contributed to one or more components. A slight difference in variance was seen in components over time (Table 2). The first component consisted of maximum sedentary bout length, weighted median sedentary bout length, fragmentation index, mean LPA time, mean sedentary time in bouts ≥5, 30, and 60 min, and mean SB. The second component included mean MVPA, and mean MVPA in bouts ≥10 min. The third component consisted of maximum sedentary bout length, weighted median sedentary bout length, and fragmentation index.

**Table 2:** Explained variances of compressed components on all four time points

Component		T1	T2	T3	T4
1	Maximum sedentary bout length Weighted median sedentary bout length Fragmentation index Mean LPA time Mean sedentary bouts ≥5 min. Mean sedentary bouts ≥30 min. Mean sedentary bouts ≥60 min. Mean sedentary time	57.3%	56.9%	53.3%	51.1%
2	MVPA MVPA bouts ≥10 min.	16.7%	17.0%	18.1%	17.3%
3	Maximum sedentary bout length Weighted median sedentary bout length Fragmentation index	10.9%	8.5%	10.1%	11.3%
	Total variance	85.9%	82.5%	81.5%	79.7%

*T1* three weeks after discharge, *T2* six months after discharge, *T3* one year after discharge, *T4* two years after discharge, *LPA* light physical activity, *MVPA* moderate-vigorous physical activity.

Distances of cluster means were equal between all four time points (P 0.713). A visual check of the components’ distribution between all four time points suggested movement behavior patterns remain relatively similar over time. Additional file 2 presents scatterplots of components’ distribution on all four time points.

At baseline, sedentary exercisers spent significantly more time in MVPA per day (9.2%) than sedentary movers and prolongers. Sedentary movers performed more time in LPA per day (33.6%) than sedentary exercisers and prolongers. Sedentary prolongers were more sedentary (77%) in prolonged bouts than sedentary exercisers and movers (Table 1).

Movement behavior variables per pattern for all four time points are presented in Table 3. Differences in movement behavior variables between patterns two years after discharge were relatively similar to baseline. Movement behavior variables between patterns at baseline and two years after discharge are presented in Additional file 3.

**Table 3:** Movement behavior variables per patterns over time

		T1	T2	T3	T4
Sedentary exercisers		*N* = 44	*N* = 47	*N* = 34	*N* = 38
	Sedentary time (hours/day)	8.97 [8.49–9.45]	8.97 [8.53–9.40]	8.84 [8.36–9.32]	9.36 [8.90–9.81]
	Sedentary%	63.6%	61.8%^4^	60.9%^4^	66.3%^2,3^
	LPA (hours/day)	3.78 [3.43–4.14]	3.82 [3.54–4.10]	3.53 [3.08–3.99]	3.36 [3.01–3.71]
	LPA%	26.9%	26.9%	26.4%	22.7%
	MVPA (hours/day)§	1.31 (0.33)^2,3^	1.56 (0.91)^1^	1.57 (0.94)^1^	1.49 (0.51)
	MVPA%§	9.2%^3^	11.0%	11.6%^1^	10.3%
	Sedentary bouts ≥5 min. (hours/day)	5.90 [5.52–6.27]	5.90 [5.51–6.29]	5.88 [5.45–6.31]	6.47 [6.11–6.84]
	Sedentary bouts ≥30 min. (hours/day)	3.18 [2.87–3.49]^4^	3.32 [3.00–3.64]^4^	3.39 [2.98–3.80]^4^	4.25 [3.78–4.71]^1,2,3^
	Sedentary bouts ≥60 min. (hours/day)	1.28 (1.16)^4^	1.38 (1.33)^4^	1.43 (1.43)^4^	2.0 (1.47)^1,2,3^
	MVPA bouts ≥10 min. (hours/day)	0.60 (0.37)	0.65 (0.47)	0.65 (0.64)	0.67 (0.30)
Sedentary movers		*N* = 90	*N* = 96	*N* = 99	*N* = 84
	Sedentary time (hours/day)	8.4 [8.11–8.72]	8.66 [8.35–8.98]^4^	8.61 [8.28–8.94]	8.10 [7.72–8.49]^2^
	Sedentary%	63.1%^4^	61.8%	63.2%	59.6%^1^
	LPA (hours/day)	4.57 [4.26–4.89]	4.80 [4.49–5.11]	4.48 [4.16–4.80]	5.04 [4.68–5.40]
	LPA%	33.6%	34.2%	32.6%	35.6%
	MVPA (hours/day)§	0.44 (0.49)^2,3^	0.59 (0.43)^1^	0.59 (0.66)^1^	0.50 (0.61)
	MVPA%§	2.9%	4.4%	4.2%	3.5%
	Sedentary bouts ≥5 min. (hours/day)	5.50 [5.24–5.76]	5.72 [5.44–6.00]^4^	5.50 [5.23–5.77]	5.06 [4.74–5.37]^2^
	Sedentary bouts ≥30 min. (hours/day)	3.02 [2.82–3.23]^2^	3.28 [3.06–3.49]^1^	3.08 [2.88–3.28]	2.88 [2.64–3.13]
	Sedentary bouts ≥60 min. (hours/day)	1.30 (0.81)^2^	1.56 (0.99)^1^	1.43 (0.87)	1.30 (1.01)
	MVPA bouts ≥10 min. (hours/day)	0.06 (0.18)^2^	0.13 (0.21)^1^	0.07 (0.20)	0.10 (0.2)
Sedentary prolongers		*N* = 66	*N* = 57	*N* = 67	*N* = 78
	Sedentary time (hours/day)	10.59 [10.25–10.94]	11.02 [10.61–11.44]	10.87 [10.46–11.29]	10.76 [10.48–11.03]
	Sedentary%	77.1%	77.1%	73.8%	75.1%
	LPA (hours/day)	2.78 [2.58–2.97]	2.80 [2.53–3.08]	2.92 [2.68–3.16]	2.95 [2.70–3.20]
	LPA%	20.0%	19.7%	22.2%	21.9%
	MVPA (hours/day)§	0.41 (0.45)	0.27 (0.71)	0.45 (0.63)	0.34 (0.52)
	MVPA%§	2.5%	1.8%	3.3%^4^	2.5%^3^
	Sedentary bouts ≥5 min. (hours/day)	8.20 [7.87–8.54]	8.48 [8.07–8.88]	8.05 [7.66–8.43]	8.06 [7.76–8.35]
	Sedentary bouts ≥30 min. (hours/day)	5.88 [5.57–6.18]	6.25 [5.83–6.67]	5.96 [5.62–6.30]	5.86 [5.55–6.17]
	Sedentary bouts ≥60 min. (hours/day)	3.44 (1.34)	3.49 (1.62)	3.48 (1.29)	3.37 (1.54)
	MVPA bouts ≥10 min. (hours/day)	0.09 (0.22)	0.04 (0.22)	0.09 (0.24)	0.04 (0.21)

Data is presented as mean [confidence interval 95%], median (IQR), %

*T1* three weeks after discharge, *T2* six months after discharge, *T3* one year after discharge, *T4* two years after discharge, *LPA* light physical activity, *MVPA* moderate-vigorous physical activity

^1^ = significant difference with T1

^2^ = significant difference with T2

^3^ = significant difference with T3

^4^ = significant difference with T4

§ = transformation with square root, back transformed for mean [95%CI] or median (IQR)

### Individuals changes their movement behavior pattern

**Fig. 2:**
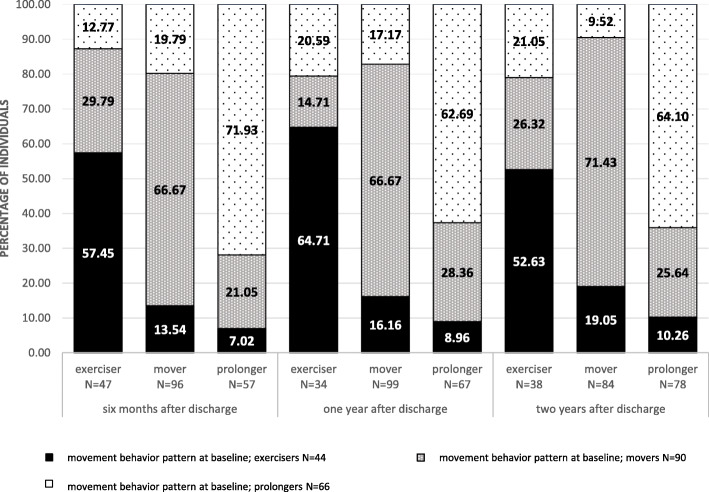
Individuals’ change in movement behavior patterns within the first two years after discharge to home

At baseline, there were 44 (22%) *sedentary exercisers,* 90 *(45%) sedentary movers,* and 66 (33%) *sedentary prolongers.* Movement behavior patterns of individuals during the two years is visualized in Fig. 2. People that changed their movement behavior pattern from sedentary exerciser to sedentary mover, spent significant less time in MVPA and LPA. To allocate from sedentary mover to sedentary prolonger people were significant less physically active in LPA and MVPA, and were significantly more sedentary. People that changed their movement behavior pattern from sedentary mover to sedentary prolonger spent significant less time in LPA and significant more time in SB. In total, 70 individuals (35%) changed the composition of their movement behavior during waking hours, resulting in allocation to another movement behavior pattern during two years after discharge. Within the first two years, the proportion of sedentary exercisers and sedentary movers decreased by 3%. Sedentary prolongers increased by 6%, accounting for 39% of the study population.

Of the seventy individuals who changed their movement behavior pattern over time, 37% improved (i.e. sedentary mover to sedentary exerciser, and sedentary prolonger to sedentary mover or exerciser) and 63% deteriorated to a movement behavior pattern with higher health risks (i.e. sedentary exerciser to sedentary mover or sedentary prolonger and sedentary mover to sedentary prolonger).

The proportion of sedentary prolongers that improved their movement behavior pattern to a movement behavior pattern with less health risks increased by 12.5% within the first six months. Hereafter the proportion declined, with 0.8% in the first year and 6.6% two years after discharge.

After two years, the entire group of sedentary prolongers consisted of 64% baseline sedentary prolongers, 26% sedentary movers, and 10% sedentary exercisers.

## Discussion

This study demonstrates the composition of movement behavior patterns in people with a first-ever stroke remains stable during the first two years after home discharge. However, individuals’ changes in movement behavior pattern over time. Of the 200 participants, 35 % changed their movement behavior pattern during the two-year follow-up. Two years after a first-ever stroke, 40% of the people who changed their pattern, deteriorated to sedentary prolonger. The proportion of sedentary prolongers, the most unfavorable movement behavior pattern, increased to 39% over time.

This study was in the sequel of the research of Wondergem et al. [6] In a cross-sectional study Wondergem et al. identified three movement behavior patterns in people with stroke [6]. To validate these results and investigate if these movement behavior patterns remain similar over time, movement behavior patterns were identified at four time points from 3 weeks to two years after home discharge. The similar patterns at all four time points showed that movement behavior patterns remain similar up to two years after a first identical stroke. If this study identified other patterns at later time points, the movement behavior patterns were probably unusable in daily practice.

In line with studies investigating single aspects, we found movement behavior patterns remain stable over time [11, 17, 18]. The importance of addressing movement behavior in patterns instead of single aspects is supported by former research in movement behavior and health outcomes [8, 13, 15, 50]. Earlier research described that one movement behavior aspect (sufficient amounts of MVPA) could be insufficient to compensate for the other aspect’s health risks (high and prolonged SB) [11]. Additionally, health risks could be amplified by each other. Maddison et al. [51] showed that individuals who were highly sedentary and less physically active had a substantially higher mortality rate (RR 7.8) compared to individuals with only high sedentary time (RR 2.0) or low PA (RR 2.0). Replacing 30 minutes of SB with LPA or MVPA could lower the mortality risk [52].

Within two years after discharge, individuals changed their movement behavior pattern. Most improvements were seen between baseline and six months after home discharge. Hereafter, movement behavior of sedentary exercisers and sedentary movers deteriorated to a movement behavior pattern with higher health risks. Similar with the period that improvement was seen in this study, other studies investigating single aspects of movement behavior reported an increase in physical activity in the first three to six months after stroke [50, 53]. An increase in physical activity could be the effect of improvement in physical functioning after stroke [50]. However, our results reflect that people with stroke are not able to maintain their movement behavior over the following years. Mahendran et al. [18] found preliminary evidence that people with stroke shorten the duration of physical activity bouts three months after discharge. It could be hypothesized that after discharge to home people try to adapt physical activities, trained during the therapy sessions, into their own environment [50]. However, it is difficult to precieve behavioral change and maintain a healthy lifestyle [54]. Therefore, it is important that people with stroke are supported to improve their movement behavior by health care professionals, using interventions targeting behavioral change, starting directly in the acute phase [53, 54].

The proportion of sedentary prolongers increased significantly over time. Because of their highly sedentary (10.8 h/day) and inactive lifestyle, sedentary prolongers are the most unfavorable movement behavior pattern [51, 55]. A further deterioration in movement behavior over time could be expected. On the one hand, because of aging [56, 57], however, stroke-related factors (e.g. stroke related impairments, embarrassment, fear of recurrent stroke) influence individuals’ uptake in PA and time in SB [11, 53, 58]. This reflects people with stroke could be more at risk for secondary complications, especially sedentary prolongers and movers, than healthy peers. Therefore, people with stroke should be offered a personalized, tailored program based on their movement behavior pattern.

After two years, the proportion of sedentary prolongers consisted of 39% of sedentary exercisers or movers at baseline. People significantly changed their movement behavior to allocate to another movement behavior pattern over time. For example, for a sedentary exerciser or mover to become a sedentary prolonger, people have to be considerably more sedentary (2 h/day) and spend at least one hour per day less in respectively MVPA or LPA. This indicates people in this study truly changed their movement behavior resulting in allocation to another movement behavior pattern. The allocation of individuals to another movement behavior pattern raised an interesting research question. Namely, if and what variables could explain this change in individuals movement behavior pattern? Because of the relatively small proportion of people that allocate to another movement behavior pattern compared to the great amount of variables that could explain this change in individuals’ movement behavior pattern, secondary analysis was not possible. Future research could explore which variables are associated with change in individuals movement behavior and allocation to another movement behavior pattern over time. To date, we are not yet able to identify people at risk for future unhealthy movement behavior pattern. Therefore it is crucial to repeatedly measure stroke survivors’ movement behavior to provide personalized trajectories and prevent secondary complications.

A strength of this study was that LPA was included as an individual aspect of movement behavior. Most studies reported leisure-time PA as a whole. Breaking up SB with LPA could contribute to better health outcomes [10, 13, 14, 59]. Moreover, encouraging people with stroke to increase their MVPA requires more behavioral change than breaking up SB with LPA [52, 60]. Therefore, LPA might play a significant role in movement behavior interventions in people with stroke.

This study’s limitation was that we used PCA to compress ten movement behavior variables to three components and calculate z-scores for cluster analysis. This could have resulted in a small variability between the components over time. However, the total explained variance of the components on all four time points was more than the suggested 60% by Hair et al. [61]. Moreover, similar components and ultimately similar patterns as Wondergem et al. [6] were identified.

Overall, most people with stroke are inactive and highly sedentary. Two years after a first-ever stroke, a great amount of individuals significantly deteriorate their movement behavior to a movement behavior pattern with higher health risks. Therefore, preventing people with stroke from becoming sedentary prolonger or mover is essential as a way of secondary prevention. Sedentary exercisers should be encouraged to maintain their PA and reduce SB. Additionally, sedentary movers should be stimulated to improve their MVPA and decrease SB. Above all, sedentary prolongers should be supported to increase PA (MVPA and LPA), and break up and substitute their SB. At last, the increase of sedentary prolongers two years after discharge to home is of great concern. Future research is needed to explore factors associated with changes in movement behavior and prediction of movement behavior patterns in the long term.

## Conclusions

Movement behavior in people with stroke can be distinct in three movement behavior patterns. Although the compositions of movement behavior patterns in people with a first-ever stroke remain stable over time, individuals change their movement behavior resulting in allocation to another movement behavior pattern. After two years, more people deteriorated to a movement behavior pattern with higher health risks. The amount of people allocated to sedentary movers and sedentary prolongers, and the increase of people allocated to these patterns over time, are of great concern and underlines the importance of improving and maintaining a healthy movement behavior in people with stroke to prevent future health risks.

## Supplementary Information

**Additional file 1.** Overview of measurements

**Additional file 2.** Scatterplots presenting the distribution of the three components on all four time points

**Additional file 3.** Movement behavior variables between patterns at baseline and two years after discharge to home

## Data Availability

The data that support the findings of this study are available from Fontys University of Applied Sciences but restrictions apply to the availability of these data, which were used under license for the current study, and so are not publicly available. Data are however available from the authors upon reasonable request and with permission of Fontys University of Applied Sciences.

## Abbreviations

PA: Physical activity
SB: Sedentary behavior
LPA: Light physical activity
MVPA: Moderate to vigorous physical activity
