# Supplementary material for: Movement behavior patterns composition remains stable, but individuals change their movement behavior pattern over time in people with a first-ever stroke
Source: Eur Rev Aging Phys Act. 2022 Apr 22;19:11. doi: 10.1186/s11556-022-00290-4 (PMC9026674; doi:10.1186/s11556-022-00290-4)
Supplement: Supplementary file 1 — Additional file 1. Overview of measurements [file 11556_2022_290_MOESM1_ESM.docx]

***Additional files***

Additional file 1. Overview of measurements

| Outcome | Instrument | Type | T1 | T2 | T3 | T4 |
| --- | --- | --- | --- | --- | --- | --- |
| Movement behavior | Activ8 | Measuring device | X | X | X | X |
| Functional status | Stroke impact scale 3.0 | Questionnaire | X |  |  |  |
| Stroke characteristics | Hemisphere;  National institutes of Health Stroke Scale;  Date of stroke | Observational scale | X |  |  |  |
| Comorbidity | Cumulative Illness Rating Scale | Questionnaire | X |  |  |  |
| Pre stroke physical activity | Physical activity assessment | Questionnaire | X |  |  |  |
| Cognitive status | Montreal cognitive assessment | Assessment | X |  |  |  |
| Emotional status | Hospital anxiety and depression | Questionnaire | X |  |  |  |
| Fatigue | Checklist individual strength – fatigue subscale | Questionnaire | X |  |  |  |
| Self-efficacy | Self- efficacy for symptom management scale | Questionnaire | X |  |  |  |
| Demographic factors | Age  Gender  Living status | Report | X |  |  |  |
| Social support | Social Support list | Questionnaire | X |  |  |  |
| Discharge location | Discharge to home from | Report | X |  |  |  |
