# Supplementary material for: Movement behavior patterns composition remains stable, but individuals change their movement behavior pattern over time in people with a first-ever stroke
Source: Eur Rev Aging Phys Act. 2022 Apr 22;19:11. doi: 10.1186/s11556-022-00290-4 (PMC9026674; doi:10.1186/s11556-022-00290-4)

Additional file 2. Scatterplots presenting the distribution of the three components on all four time points


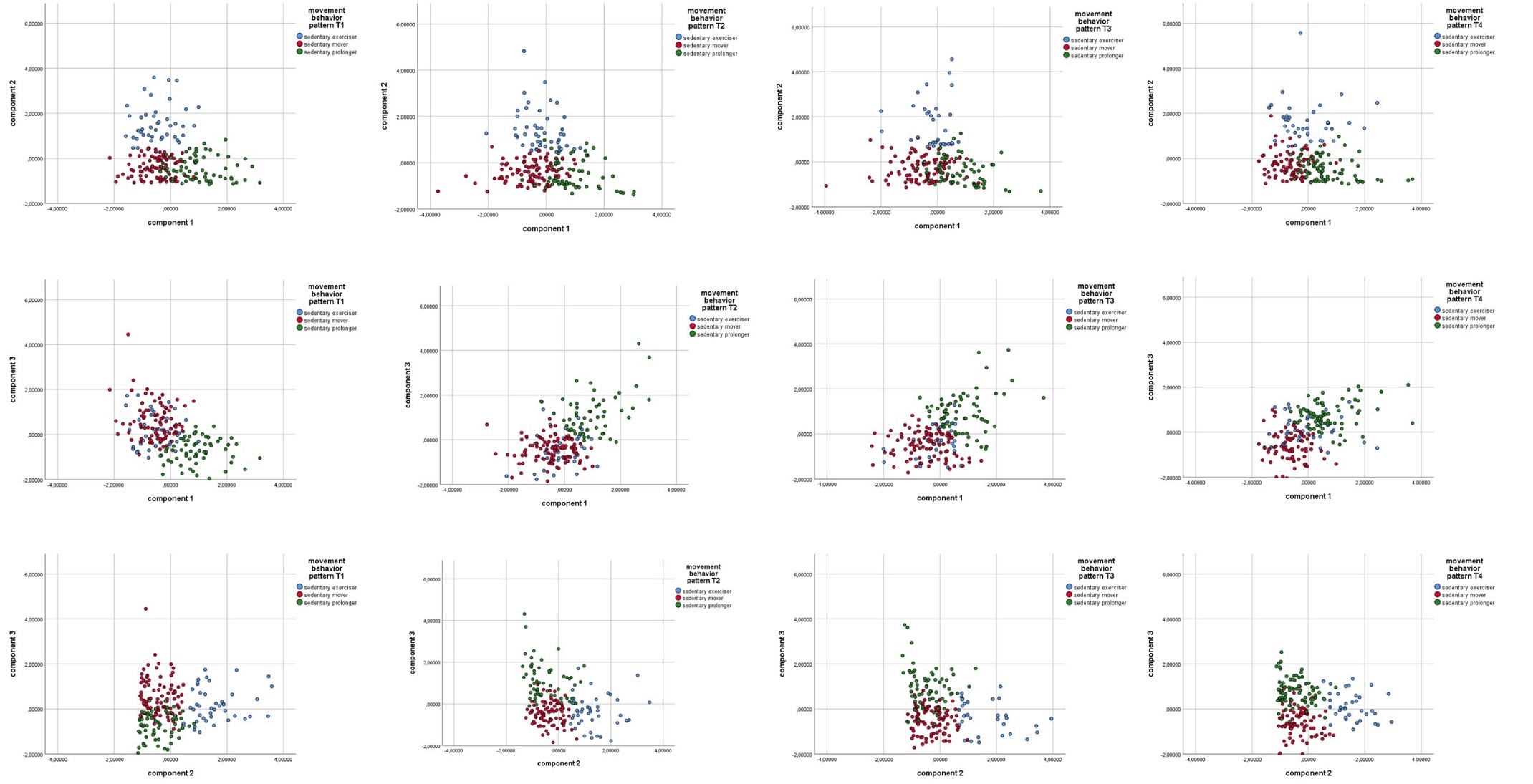

Supplement: Supplementary file 2 — Additional file 2. Scatterplots presenting the distribution of the three components on all four time points [file 11556_2022_290_MOESM2_ESM.docx]
