# Supplementary material for: Movement behavior patterns composition remains stable, but individuals change their movement behavior pattern over time in people with a first-ever stroke
Source: Eur Rev Aging Phys Act. 2022 Apr 22;19:11. doi: 10.1186/s11556-022-00290-4 (PMC9026674; doi:10.1186/s11556-022-00290-4)
Supplement: Supplementary file 3 — Additional file 3. Movement behavior variables between patterns at baseline and two years after discharge to home [file 11556_2022_290_MOESM3_ESM.docx]

Additional file 3. Movement behavior variables between patterns at baseline and two years after discharge to home

|  | T1 | | | | T4 | | | |
| --- | --- | --- | --- | --- | --- | --- | --- | --- |
| *Movement behavior* | Total group  N=200 | Sedentary exercisers  N=44 | Sedentary movers  N=90 | Sedentary prolongers  N=66 | Total group  N=200 | Sedentary exercisers  N=38 | Sedentary movers  N=84 | Sedentary prolongers  N=78 |
| SB time (hours)  SB % | 9.3 [9.0-9.5]  67.8% | 9.0 [8.5- 9.5]^‡^  63.6% | 8.4 [8.1-8.7] ^‡^  63.1% | 10.6[10.3-10.9]^*†^  77.1% | 9.4 [9.1-9.6]  66.9% | 9.4 [8.9-9.8] ^†‡^  66.3% | 8.1 [7.7-8.5]^*‡^  59.6% | 10.8[10.5-11.0]^*†^  75.1% |
| LPA time (hours)  LPA % | 3.8 [3.6-1.0]  27.6% | 3.8[3.4-4.1]^†‡^  26.9% | 4.6[4.3-4.9]^*‡^  33.6% | 2.8[2.6-3.0]^*†^  20.0% | 3.9 [3.7-4.1]  27.8% | 3.4[3.0-3.7]^†^  22.7% | 5.0[4.7-5.4]^*‡^  35.6% | 3.0[2.7-3.2] ^†^  21.9% |
| MVPA time (hours)  MVPA % | 0.6 (0.7)  3.9% | 1.3 (0.3) ^†‡^  9.2% | 0.4 (0.5)^*^  2.9% | 0.4 (0.5)^*^  2.5% | 0.5 (0.9)  3.9% | 1.5 (0.5) ^†‡^  10.3% | 0.5 (0.6)^* ‡^  3.5% | 0.3 (0.5)^*†^  2.5% |
| Sedentary bouts ≥ 5 min. (hours) | 6.5 [6.2-6.7] | 5.9[5.5-6.3] ^‡^ | 5.5[5.2-5.8] ^‡^ | 8.2[7.9-8.5]^*†^ | 6.5 [6.2-6.8] | 6.5[6.1-6.8]^†‡^ | 5.1[4.7-5.4]^*‡^ | 8.1[2.7-3.2]^*†^ |
| Sedentary bouts ≥ 30 min (hours) | 4.0 [3.8-4.2] | 3.2[2.9-3.5] ^‡^ | 3.0[2.8-3.2] ^‡^ | 5.9[5.6-6.2]^*†^ | 4.3 [4.5-4.6] | 4.3[3.8-4.7]^†‡^ | 2.9[2.6-3.1]^*‡^ | 5.9[5.6-6.2]^*†^ |
| Sedentary bouts ≥ 60 min (hours) | 1.2 (1.8) | 1.3 (1.2) ^‡^ | 1.3 (0.8) ^‡^ | 3.4 (1.3)^*†^ | 2.2 (2.0) | 2.0 (1.5) ^†‡^ | 1.3 (1.0)^*‡^ | 3.4 (1.5)^*†^ |
| MVPA bouts ≥10 min. (hours) | 0.1 (0.3) | 0.6 (0.4) ^†‡^ | 0.1(0.2)^*^ | 0.1(0.2)^*^ | 0.1(0.3) | 0.7 (0.3) ^†‡^ | 0.1 (0.2)^*^ | 0.0 (0.2)^*^ |
| WMSB (min) | 22.5 [20.6-24.3] | 15.8[13.4-18.6] ^‡^ | 15.8[14.2-17.3] ^‡^ | 36.2[33.0-39.3]^*†^ | 24.8[22.3-27.3] | 23.7[18.5-28.9] ^†‡^ | 14.5[12.7-16.3]^*‡^ | 36.4[32.1-40.7]^*†^ |
| Maximum sedentary bout length (min.) | 135.0 [128.3-141.7] | 120.5 [108.9-132.2] ^‡^ | 115.2 [108.9-121.6] ^‡^ | 171.6 [158.6-184.7]^*†^ | 144.5 [139.4-149.7] | 148.1 [137.1-159.2] | 127.4 [119.9-134.9] ^‡^ | 127.4 [153.8-168.7] ^†^ |
| Fragmentation index | 1.9[1.9-2.0] | 2.1 [2.0-2.1]^‡^ | 2.0 [2.0-2.1]^‡^ | 1.6[1.6-1.7]^*†^ | 1.8 [1.8-1.9] | 1.9 [1.8-2.0]^‡^ | 2.0 [1.9-2.0]^‡^ | 1.7[1.6-1.7]^*†^ |
| Weartime | 13.7 [13.5-13.9] | 14.1[13.7-14.5] ^†^ | 13.4[13.7-14.5]^*^ | 13.8 [13.4-14.1] | 14.0 [13.8-14.2] | 14.3 [13.9-14.7] | 14.5 [13.9-14.1] | 36.4 [13.9-14.4] |

*Variables are presented as Mean±sd, median (IQR), N(%) or Mean [95% Confidence interval]*

*IPR=inpatient rehabilitation, IPGR=inpatient geriatric rehabilitation, LPA= light physical activity, MVPA= moderate-vigorous physical activity, WMBS=Weighted Median sedentary bout length*

*^*^=significant differences with sedentary exercisers,*

^†^*= significant differences with sedentary movers,*

^‡^*= significant. differences with sedentary prolongers*
